# Physical Activity and Exercise Patterns of Submariners in Land and Sea Environments

**DOI:** 10.1093/milmed/usaf415

**Published:** 2025-08-14

**Authors:** Benjamin Kirk, Georgios Mavropalias, Anthony Blazevich, Jodie Cochrane Wilkie, Aus Molan, Kazunori Nosaka

**Affiliations:** School of Medical and Health Sciences, Edith Cowan University, Joondalup, WA 6027, Australia; Performance and Expertise Research Centre, Macquarie University, Macquarie Park, NSW 2113, Australia; Biomechanics, Physical Performance, and Exercise Research Group, Department of Health Sciences, Macquarie University, Macquarie Park, NSW 2113, Australia; School of Medical and Health Sciences, Edith Cowan University, Joondalup, WA 6027, Australia; School of Medical and Health Sciences, Edith Cowan University, Joondalup, WA 6027, Australia; School of Medical and Health Sciences, Edith Cowan University, Joondalup, WA 6027, Australia; Physical Activity, Sport and Exercise Research Theme, Faculty of Health, Southern Cross University, Gold Coast, QLD 4225, Australia; Exercise Medicine Research Institute, Edith Cowan University, Joondalup, WA 6027, Australia; PathWest Laboratory Medicine, Murdoch, WA 6150, Australia; School of Medical and Health Sciences, Edith Cowan University, Joondalup, WA 6027, Australia

**Keywords:** Submariners, physical activity, sedentary behavior, exercise barriers, sleep quality, operational readiness

## Abstract

**Introduction:**

Submarine environments pose unique challenges to maintaining physical activity and exercise routines due to confined spaces, demanding schedules, and limited resources. This study investigated submariners’ physical activity patterns, sleep quality, and perceived exercise barriers in both land- and sea-based settings, with the goal of informing targeted health interventions.

**Materials and Methods:**

Ethics approval was granted by the Defence Science and Technology Group and Edith Cowan University review panels. This cross-sectional study surveyed 21 Royal Australian Navy submariners (25-48 years; 18 male) using a modified version of the International Physical Activity Questionnaire. Participants reported weekly engagement in general physical activity (e.g., walking, moderate- and vigorous-intensity activity) and structured exercise training (high-intensity interval training, moderate-intensity continuous training, and resistance training [RT]), as well as sitting time and sleep duration. Additional items assessed perceived barriers and motivations to exercise, time spent on land and at sea over the past 12 months (9 ± 3 and 3 ± 3 months, respectively), and their longest continuous deployment. Descriptive statistics and paired *t*-tests were used to compare outcomes between land and sea environments.

**Results:**

Total physical activity was lower (*P* < .001) at sea (118 ± 30 minutes/week) compared to land (745 ± 60 minutes/week), with the greatest reductions observed in walking (−86%, *P* < .001) and moderate-intensity cardiovascular training (−95%, *P* = .002). High-intensity interval training declined by 81% (*P* = .006), and RT dropped by 84% (*P* = .045). Reported barriers at sea included water usage restrictions (57%), limited space (43%), inadequate facilities (43%), time constraints (38%), fatigue (38%), and noise restrictions (19%). Sleep quality declined by 37% at sea (*P* < .001), though changes in sleep duration were not statistically significant. Sitting time increased by 51% on workdays (*P* = .014).

**Conclusions:**

Sea deployments are associated with substantial declines in physical activity and sleep quality among submariners, accompanied by increased sitting time and widespread behavioral disengagement. These findings highlight the need for practical strategies to support health, well-being, and operational readiness in constrained environments. Strengths of the study include context-specific survey design and rich participant engagement, as reflected by detailed qualitative responses. Limitations include small sample size, variability in responses, potential recall bias, and the inability to assess survey reliability because of ethical constraints. Future research should prioritize longitudinal designs and explore implementable interventions to promote physical activity and sleep during deployment. These findings may also apply to other constrained environments, such as naval surface ships, remote field sites, or mining operations, where similar occupational barriers exist. Overall, these results offer valuable insight into how deployment conditions shape health behaviors in submariners and provide a foundation for developing evidence-based strategies to improve activity and well-being in this and similar populations.

## INTRODUCTION

Physical fitness is a key component of occupational capability, particularly in high-demand military environments, such as submarines, where personnel must perform physically strenuous tasks requiring aerobic and neuromuscular endurance.^1^ In addition to supporting operational readiness, regular physical activity reduces the risk of chronic diseases such as type II diabetes and cardiovascular disease,^2–4^ lowers musculoskeletal injury risk,^5^^,^^6^ and contributes to fewer sick days, reduced medical costs, and a lower risk of discharge due to injury or illness.^7^ It is also associated with improved resilience; an essential trait in military contexts.^8^

Regular physical activity further supports mental health and cognitive function, enhancing mood, attention, and decision-making.^9–12^ Thus, both structured exercise and general physical activity are important not only for maintaining physical health but also for sustaining well-being and performance during extended deployments. However, it remains unclear how submariners adapt their activity levels at sea compared to on land.

Current activity guidelines recommend that adults engage in 150-300 minutes of moderate-intensity or 75-150 minutes of vigorous-intensity activity weekly, or an equivalent combination, with additional recommendations for muscle-strengthening activities and reduced sedentary time.^13^^,^^14^ Yet fewer than 20% of Australian adults meet these targets, with similar trends observed globally.^15^^,^^16^ These recommendations are difficult to meet under typical conditions and likely even more so in extreme occupational environments.

Submarine operations involve prolonged periods in confined, isolated conditions with limited space for exercise.^17^ Environmental stressors, including elevated carbon dioxide (CO_2_),^18^ lack of natural light, artificial atmospheres,^19^ calorie-dense food,^20^^,^^21^ limited water for hygiene practices, and rotating shift schedules, further challenge routine health behaviors. These constraints may contribute to reduced physical activity while deployed, with potential negative consequences for both short- and long-term health.

Given these challenges, it is important to understand how submariners adapt their activity habits during deployments. This information could guide the development of evidence-based strategies to support physical activity in this unique environment. Therefore, the aim of this study was to examine the amount and type of physical activity and exercise performed by Royal Australian Navy submariners on land and at sea, and to identify key barriers affecting participation in both settings.

## METHODS

### Overview

Submariners with experience in both onshore and at-sea duties across their career were recruited to complete an online survey. Ethical approval was granted by the Defence Science and Technology Low Risk Ethics Panel (MD 02-22) and the Edith Cowan University Human Research Ethics Committee (2022-03625-KIRK). The Defence Science and Technology Group facilitated survey distribution. Participants provided informed consent electronically. Thirty-three began the questionnaire; 22 completed it fully. One was excluded for reporting an implausible 44 hours of exercise per week during deployment, leaving a final sample of 21 submariners.

### Survey

The survey was adapted from the International Physical Activity Questionnaire (IPAQ), typically used to assess physical activity over the past 7 days. For this study, it was modified to capture recalled physical activity and exercise during participants’ most recent land and sea postings, allowing contextual comparison without a fixed timeframe. The survey was administered via Qualtrics (Qualtrics, United States).

Participants reported time spent on land and at sea in the past 12 months, along with the duration of their longest continuous submarine deployment. Physical activity was categorized as general daily activity or dedicated exercise training. General activity included vigorous (e.g., heavy lifting), moderate (e.g., carrying light loads), and walking. Exercise training included high-intensity interval training (HIIT; e.g., sprints, cycling intervals), moderate-intensity continuous training (MICT; e.g., steady jogging/cycling), and resistance training (e.g., weighted or bodyweight exercises). Follow-up questions confirmed alignment with these definitions.

Participants also reported sitting and sleeping time in both environments. A 5-point Likert scale (“Strongly Agree” to “Strongly Disagree”) assessed exercise enjoyment. Additional questions explored exercise motivations (e.g., health, personal improvement, fitness assessments, peer influence, or competition). Open-text fields allowed participants to describe other motivations, as well as barriers, sleep, hygiene, and diet-related issues, and suggest potential improvements.

### Data Analyses

Weekly general activity was calculated based on Australian physical activity guidelines, where vigorous activity is doubled and summed with moderate activity and walking.^22^ This same weighting was applied to HIIT when calculating total exercise training. Final scores were derived as:


$$\begin{array}{c} \text{General}\;\text{physical}\;\text{activity}=(\text{vigorous}\;\text{activity}\times 2)+\text{moderate}\;\text{activity}+\text{walking}\\ \text{Total}\;\text{activity}=[(\text{vigorous}\;\text{activity}\times 2)+\text{moderate}\;\text{activity}+\text{walking}]+[(\text{HIIT}\times 2)+\text{MICT}+\text{RT}] \end{array}$$

Open-ended responses were analyzed thematically. Recurring themes were identified and tallied as a percentage of the total sample. For example, answers to “Are there any barriers that reduce your ability to perform regular physical activity/exercise whilst at sea?” were grouped into categories (e.g., environmental or logistical constraints).

### Statistical Analyses

Descriptive statistics (mean, SD, and frequency) were used to summarize responses. Normality was assessed using the Shapiro-Wilk test. Paired *t*-tests and Cohen’s *d* effect sizes were used to assess changes in the criterion measures between land and sea. Wilcoxon signed-rank tests and rank-biserial correlations were applied for non-normal data. Effect sizes were interpreted as follows: for Cohen’s *d*, 0.1-0.2 was considered trivial, 0.2-0.5 small, 0.5-<0.8 moderate, and >0.8 large; for rank-biserial correlations, 0.1 was considered small, 0.3 medium, and >0.5 large. These categories are conceptually similar, with rank-biserial values reflecting ranked differences rather than standardized mean differences. Statistical significance was set at *P* < .05. All analyses were conducted using Jamovi (version 2.3.21, Jamovi Project, 2018). Data are reported as mean ± SD.

## RESULTS

### Participants

Participants were predominantly male (*n* = 18/21) with an age of 36 ± 7 years (range: 25-48). Participants reported spending 3 ± 3 months at sea and 9 ± 3 months on land in the previous 12 months. The longest continuous deployment was 4.1 ± 4.5 months (range: 0.8-18). Nineteen participants had recent sea time; 2 reported only land duties in the past year but had prior deployment experience.

### Physical Activity

Vigorous activity participation declined from 10 participants on land to 3 at sea, though this was not statistically significant (*P* = .074). In contrast, moderate activity and walking time significantly decreased during deployment. Moderate activity dropped by 79 ± 72% (*P* = .004, *r_rb_* = 0.92), with participation falling from 14 to 8. Walking declined by 86 ± 60% (*P* < .001, *d *= 1.08), with only 5 participants walking at sea versus 21 on land.

High-intensity interval training time declined by 81 ± 55% (*P* = .006, *r_rb_* = 0.87), with participation decreasing from 13 to 2. Moderate-intensity continuous training fell by 95 ± 82% (*P* = .002, *r_rb_* = 0.90), with only 1 participant training at sea compared to 16 on land. Resistance training time dropped by 84 ± 71% (*P* = .045, *r_rb_* = 0.67), with participation falling from 9 to 4. **Table 1** summarizes participant changes.

**Table 1.** Activity on Land and Sea Measured in Minutes Per Week

	**Average for all submariners** **(minutes per week)**	**Number of active submariners** **(*n* = 21)**	**Average for active submariners** ** (minutes per week)**
**Land activity**			
Vigorous	24 ± 32	10	50 ± 29
Moderate	79 ± 107	14	119 ± 113
Walking	380 ± 330	21	NA
High-intensity cardiovascular training	48 ± 66	13	77 ± 69
Moderate-intensity cardiovascular training	94 ± 127	16	124 ± 132
Resistance training	49 ± 80	9	113 ± 88
General physical activity	506 ± 352	21	NA
Total activity	745 ± 432	21	NA
**Sea activity**			
Vigorous	8 ± 22	3	55 ± 31
Moderate	16 ± 30^a^	8	43 ± 36
Walking	54 ± 130^a^	5	228 ± 188
High-intensity cardiovascular training	9 ± 29^a^	2	98 ± 11
Moderate-intensity cardiovascular training	5 ± 23^a^	1	105
Resistance training	8 ± 24^a^	4	41 ± 43
General physical activity	86 ± 139^b^	10	341 ± 110
Total activity	118 ± 177^b^	11	408 ± 119

Number of active submariners represents participants who completed more than zero minutes of activity per week. Active average represents the average time spent by active participants performing that activity.

Values are mean ± SD. NA all participants were active.

a Significant (*P* < .05) difference from sea.

b *P* < .001 difference from land activity.

**Figure 1.**
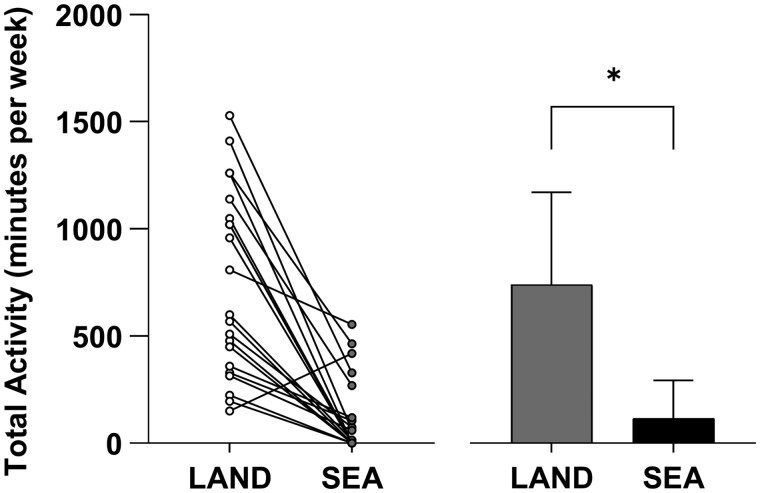
Individual (left) and mean ± SD (right) total physical activity time (minutes per week) on land and at sea. *Significant (*P* < .05).

General physical activity volume during sea deployment decreased by 83 ± 60% (*P* < .001, *d *= 1.29), with the number of active participants falling from 21 on land to 10 at sea. Total activity, combining general activity and exercise training, declined by 84 ± 59% (*P* < .001, *d *= 1.44), with active participants dropping from 21 to 11 during deployment. Individual data are shown in **Figure 1**.

### Sitting Time

Time spent sitting on workdays increased by 51 ± 78% during sea deployment (*P* = .014, *d *= 0.61), while no significant change was observed on non-workdays (*P* = .638, *d *= 0.11) (**Table 2**).

**Table 2.** Inactivity on Land and at Sea, Measured in Hours Per Day

Inactivity	Land	Sea
Sitting—work day (hours)	6.1 ± 2.3	9.2 ± 4.1^a^
Sitting—non-work day (hours)	4.6 ± 3.8	4.2 ± 3.8
Sleep (hours)	7.4 ± 1.1	6.6 ± 1.4
Completed in one sleep period (number of submariners)	19/20	2/19^a^
Sleep quality (%)	77 ± 11	49 ± 21^a^

Values are mean ± SD.

a Significant (*P* < .05) difference from land activity.

### Sleep

Sleep duration declined from 7.4 ± 1.1 hours on land to 6.6 ± 1.4 hours at sea, though this was not statistically significant (*P* = .118, *d *= 0.366). Sleep quality, however, dropped significantly by 37%, from 77 ± 11 to 49 ± 21 (*P* < .001, *d *= 1.11). Most participants reported single-session sleep on land and split sleep at sea (**Table 2**).

### Exercise Enjoyment, Motivations, and Barriers

Exercise enjoyment declined at sea, with mean agreement falling from 80 ± 19% on land to 50 ± 25% at sea. Motivations also differed notably between environments. On land, participants exercised primarily for health (90%) and personal improvement (67%), with fewer citing sports competitiveness (24%), peer influence (10%), mental health (5%), or passing the Basic Fitness Assessment (BFA) (5%); 10% did not exercise. At sea, these motivations declined sharply: 33% cited health, 10% personal improvement, and 5% either competition or the BFA. No participants cited peer influence or mental health, and 62% reported not exercising at all.

Reported barriers to physical activity varied by setting. On land, 33% cited work constraints (e.g., time, inconsistent schedules), and 10% mentioned issues with facilities or equipment. At sea, the most common barriers were water restrictions (57%), lack of space/equipment (43%), fatigue (38%), limited time (38%), noise (19%), and concerns about high-calorie food availability and potential weight gain (29%).

Participants also offered strategies to address these challenges. On land, suggestions included fitness time during work hours (14%), better gym access (10%), and reduced workload (10%). At sea, suggestions included a workout app or guide (5%), reduced work demands (5%), larger team sizes to allow a 3-person rotation (5%), and improved food quality, education, and access to supplements (10%). A detailed summary is presented in **Table 3**.

**Table 3.** Overview of Submariners’ (*n* = 21) Reported Motivations and Barriers to Physical Activity on Land and at Sea

Category	Item	Land	%	Sea	%
Motivations	Win competitions/sport	5	24	1	5
	Personal improvement	14	67	2	10
	Maintain health	19	90	7	33
	Keep up with Peers	2	10	0	0
	Just to pass BFA	1	5	1	5
	I don’t exercise	2	10	13	62
	Other	1	5	0	0
Barriers	Lack of time	7	33	11	38
	Equipment and facilities	2	10	9	43
	Fatigue	–	–	8	38
	Hygiene (showering)	–	–	10	57
	Interrupting others (noise)	–	–	4	19

Abbreviation: BFA, Basic Fitness Assessment.

## DISCUSSION

This study examined the physical activity and exercise behaviors of submariners on land and at sea. The primary finding was a substantial reduction in physical activity during sea deployments. This decline is likely driven by the considerable barriers reported by participants, including limited space, inadequate facilities, noise restrictions, time constraints, fatigue, sleep disruption, and hygiene challenges, all of which contributed to many submariners reducing or ceasing activity while deployed (**Figure 1**).

Total physical activity decreased from 745 minutes (12.4 hours) per week on land to 118 minutes (2 hours) at sea, falling below the Australian activity guideline of at least 150 minutes of moderate-intensity activity per week.^22^ This decline was primarily driven by reductions in walking and MICT, with walking decreasing by 326 minutes (from 380 to 54 minutes) and MICT by 89 minutes (from 94 to 5 minutes).

Vigorous activity, particularly HIIT, is known to improve aerobic capacity, metabolic health, and muscular function, often with a lower time demands than MICT.^23^ It is also reported as more enjoyable in some populations.^23^ Despite these benefits, HIIT participation declined from 26 minutes (13 participants) on land to 5 minutes (2 participants) at sea. Vigorous aerobic activity similarly declined from 24 minutes (10 participants) to 8 minutes (3 participants), although this change was not statistically significant. Although HIIT may be well suited to time- and space-restricted environments, low uptake ­suggests that practical barriers, such as increased sweat, hygiene limitations, and lack of privacy, may outweigh its perceived convenience. Adapting high-intensity protocols to better suit this context could be a promising avenue for future research.

Resistance training participation also declined, from 113 minutes (9 participants) on land to 41 minutes (4 participants) at sea, falling below recommendations for muscle-strengthening activity at least twice weekly.^22^ Although brief periods of inactivity may not substantially affect muscle strength or mass,^24^ previous studies have reported lean mass losses following deployments of 4 weeks or more, particularly on diesel-electric submarines.^25–28^ These effects may be because of both limited training opportunities^25^ and suboptimal atmospheric control leading to elevated CO_2_ exposure.^18^^,^^29^

Beyond health implications, sustained reductions in physical activity may impair operational performance by affecting concentration, decision-making, and team cohesion, all of which are critical in the confined and high-stakes submarine environment.^9^^,^^12^ These broader consequences highlight the need for targeted interventions that preserve both health and mission readiness throughout deployment. Understanding these patterns requires closer examination of the barriers submariners face in maintaining regular exercise at sea.

Barriers to physical activity were identified in both environments, with open-ended responses highlighting the scale and complexity of the issue. On land, 33% of submariners cited time constraints, and 10% noted limitations in equipment and facilities. Unlike civilian workplaces, submariners are often on standby, reducing opportunities to exercise during perceived “free” time. These barriers mirror those experienced by shift workers, whose irregular hours and job demands similarly impede regular exercise participation.^30^ At sea, these challenges were more pronounced, with 57% citing water restrictions that limited showering and laundry opportunities, 43% inadequate equipment and facilities, 38% time constraints, and 38% fatigue from intensive workloads and poor sleep. Noise minimization, required tactically and to avoid disturbing sleeping crew, was another notable barrier (19%). These findings align with Ponton et al,^31^ who reported dissatisfaction with space and crowding as key exercise barriers in submarine environments. Together, these operational challenges likely contributed to the 62% of submariners who reported not exercising while at sea.

A notable finding of the study was the decline in exercise enjoyment, which dropped from a mean agreement of 80 ± 19 on land to 50 ± 25 at sea. This mirrored reductions in exercise motivation, where 67% exercised for personal improvement and 90% for health on land, compared to just 10% and 33%, respectively, at sea. This decline may partly explain why 62% of submariners reported no exercise at sea, versus only 10% on land. Given the strong link between enjoyment and adherence,^32^ strategies that promote variety or social support may improve engagement. One participant remarked “exercise at sea must be seen as a requirement as opposed to a luxury or privilege” suggesting it is not sufficiently prioritized during deployments. Therefore, reframing physical activity as essential to operational readiness, rather than optional, may help normalize it as a daily practice. These findings highlight the need to position regular exercise as an operational necessity rather than a personal choice.

Chronic sleep loss is another critical concern. In this study, sleep duration declined from 7.4 ± 1.1 hours on land to 6.6 ± 1.4 hours at sea. Although not statistically significant, sleep durations under 7 hours are associated with negative health outcomes, including metabolic dysfunction and muscle loss.^33^^,^^34^ The general population’s average sleep duration is 7.8 ± 0.9 hours,^35^ indicating that submariners, particularly while deployed, are likely at elevated risk.^36^ Fragmented sleep because of 6-hour rotating shifts likely exacerbates this risk; 17 of 19 participants reported splitting sleep into 2 sessions at sea. This pattern likely contributed to the 37% decline in sleep quality.

Workday sitting time increased at sea by 51 ± 78% (*P* = .014), while non-workday sitting time remained unchanged. One explanation is that non-workdays at sea may include time outside the submarine during resupply or rest breaks, resembling land-based conditions. Supporting this, average non-workday sitting time was similar on land (4.6 ± 3.8 hours) and at sea (4.2 ± 3.8 hours). Regardless, prolonged sitting is a concern, as it is linked to cardiovascular disease, obesity, and musculoskeletal issues.^37^^,^^38^ Future studies should examine simple interventions to encourage movement and better understand the context shaping sedentary behavior during deployments.

Participants reported spending an average of 3 ± 3 months at sea annually, raising concerns about the cumulative impact on health and performance. This underscores the need for feasible, effective strategies to support health during deployments. Future research should explore training programs adapted to space and time constraints, education on physical activity benefits, and leadership support. Furthermore, command endorsement and structured scheduling may help normalize exercise as part of daily submariner life.

Several limitations should be acknowledged. Large standard deviations across outcomes indicate considerable variability in physical activity, possibly reflecting individual differences in motivation, access, or opportunity. The retrospective survey design required participants to recall their most recent deployment experiences, with 2 occurring over 12 months prior, potentially affecting recall accuracy. This extended timeframe deviates from the standard 7-day recall window recommended by the IPAQ, which may have reduced the validity of some responses. Although a longitudinal design was considered to improve accuracy through real-time data collection across deployments, it was not feasible because of logistical and ethical constraints surrounding long-term participant tracking. The study also could not assess test-retest reliability, as a planned 2-week follow-up survey was not approved by the Defence Science and Technology Ethics Panel because of reidentification concerns. Consequently, response consistency over time could not be verified. However, the depth of open-ended responses, particularly regarding exercise barriers, indicates strong participant engagement, supporting the findings and informing future strategies.

The sample size was relatively small (*n* = 21), and although all participants were qualified Royal Australian Navy submariners, their representativeness of the broader submariner population cannot be confirmed. Objective fitness data, such as BFA results, were also unavailable. These factors limit the generalizability of findings. However, a post hoc power analysis on the primary outcome (total physical activity) showed a large effect size (*d *= 1.44) and high statistical power (1-β = 0.995), suggesting sufficient sensitivity to detect meaningful differences. Future studies should replicate these findings in larger samples and include objective physical performance data to further strengthen the evidence base. Lastly, although this study focused on submariners, the findings may also apply to personnel in other constrained or isolated environments, such as naval vessels, remote outposts, or resource extraction industries (e.g., mining or offshore platforms). These settings face similar challenges related to space, equipment access, and operational demands, and may benefit from similar strategies.

Overall, submariners experience a substantial decline in physical activity during sea deployments because of environmental, operational, and logistical constraints. This study identified significant reductions in both the volume and intensity of physical activity at sea compared to on land, alongside behavioral shifts and barriers contributing to reduced exercise participation. These findings highlight an urgent need for tailored strategies to support submariners’ physical activity, health, and performance during deployments. Addressing key barriers, such as limited space, poor sleep, hygiene restrictions, and insufficient command support can help preserve health and operational readiness. Structured programs, environmental adjustments, and leadership engagement may help reframe exercise as a core part of the daily routine. With targeted support, submariners’ physical and psychological well-being can be better sustained, ultimately contributing to safer and more effective operations.

## Data Availability

All data generated or analyzed during this study are included in this published article.

## INSTITUTIONAL CLEARANCE

This research was conducted through Edith Cowan University and approved by both the University’s Human Research Ethics Committee and the Defence Science and Technology Group Low Risk Ethics Panel. No institutional restrictions on publication apply.
